# *In silico* analysis of the molecular machinery underlying aqueous humor production: potential implications for glaucoma

**DOI:** 10.1186/2043-9113-3-21

**Published:** 2013-10-28

**Authors:** Sarah F Janssen, Theo GMF Gorgels, Peter J van der Spek, Nomdo M Jansonius, Arthur AB Bergen

**Affiliations:** 1 Department of Clinical and Molecular Ophthalmogenetics the Netherlands Institute for Neuroscience (NIN), Royal Netherlands Academy of Arts and Sciences (KNAW), Amsterdam, the Netherlands; 2 Department of Bioinformatics, Erasmus University Medical Center, Rotterdam, the Netherlands; 3 Department of Ophthalmology, University Medical Center Groningen, University of Groningen, Groningen, the Netherlands; 4 Department of Ophthalmology Academic Medical Center, Amsterdam, the Netherlands; 5 Department of Clinical Genetics Academic Medical Center, Amsterdam, the Netherlands

**Keywords:** Ciliary body epithelia, Transport, Aqueous humor production, Glaucoma, Drugs

## Abstract

**Background:**

The ciliary body epithelia (CBE) of the eye produce the aqueous humor (AH). The equilibrium between the AH production by the CBE and the outflow through the trabecular meshwork ultimately determines the intraocular pressure (IOP). An increased IOP is a major risk factor for primary open angle glaucoma (POAG). This study aims to elucidate the molecular machinery of the most important function of the CBE: the AH production and composition, and aims to find possible new molecular clues for POAG and AH production-lowering drugs.

**Methods:**

We performed a gene expression analysis of the non-pigmented (NPE) and pigmented epithelia (PE) of the human CBE of post mortem eyes. We used 44 k Agilent microarrays against a common reference design. Functional annotations were performed with the Ingenuity knowledge database.

**Results:**

We built a molecular model of AH production by combining previously published physiological data with our current genomic expression data. Next, we investigated molecular CBE transport features which might influence AH composition. These features included caveolin- and clathrin vesicle-mediated transport of large biomolecules, as well as a range of substrate specific transporters. The presence of these transporters implies that, for example, immunoglobins, thyroid hormone, prostaglandins, cholesterol and vitamins can be secreted by the CBE along with the AH. *In silico,* we predicted some of the molecular apical interactions between the NPE and PE, the side where the two folded epithelia face each other. Finally, we found high expression of seven POAG disease genes in the plasma membrane of extracellular space of the CBE, namely *APOE, CAV1, COL8A2, EDNRA, FBN1, RFTN1* and *TLR4* and we found possible new targets for AH lowering drugs in the AH.

**Conclusions:**

The CBE expresses many transporters, which account for AH production and/or composition. Some of these entries have also been associated with POAG. We hypothesize that the CBE may play a more prominent role than currently thought in the pathogenesis of POAG, for example by changing the composition of AH.

## Background

The ciliary body epithelia (CBE) in the eye, consisting of the non-pigmented (NPE) and pigmented epithelia (PE), are responsible for the production of the aqueous humor (AH). This production includes passive diffusion, active transport and production of molecules from the CBE into the AH. Via the anterior chamber, the AH exits the eye trough the trabecular meshwork (TM) and the canal of Schlemm into the venous blood system. The balance between the production and outflow of AH ultimately determines the intraocular pressure (IOP).

In many physiological *in vitro* and *in vivo* animal studies, the major ion-channel and transporter proteins involved in AH production have already been identified. In short, the chain of events leading to AH production starts with the PE cells that actively take up ions at their stromal (basolateral) surface (major channels Na^+^-K^+^-2Cl^-^ symporters, Cl^-^-HCO_3_^-^ and Na^+^-H^+^ antiporters, K^+^ channels). These ions flow passively through gap junctions between the PE and NPE cell. The NPE cells actively secrete ions into the AH (major channels Na^+^-K^+^-activated ATPase, H^+^-ATPase, Na^+^-K^+^-2Cl^-^ symport, K^+^ and Cl^-^ channels). Water follows by passive diffusion through water channels (aquaporins). Together, the studies on ion-channels led to a physiological model of AH production in the CBE (reviewed in [1,2]). However, the specific genes that code for these ion channels are still unknown for many of these channels. The first aim of our current study was to investigate the human molecular background of the physiological AH production model.

Besides the ion-channels directly involved in AH production, the CBE expresses also other transporters [3,4]. These transporters may play a role in the composition of the AH, which determines a range of other functionalities previously assigned to the CBE. These are, for example, nourishment of avascular tissues in the eye (lens, cornea), neuro-endocrine signaling to the TM and other tissues and maintenance of the immune privilege of the eye. Our second aim of this study was to investigate the genetic identity of these transporters in the CBE.

The NPE and PE are folded, polarized neuroepithelial cell layers that face each other at their apical sides. At this side of the NPE and PE, secreted and plasma membrane biomolecules of both epithelia can interact. These biomolecules include signaling molecules, growth factors and extracellular matrix molecules. We are interested in the molecular characteristics of this apical interaction between NPE and PE.

In disease state, like primary open angle glaucoma (POAG), AH composition may be disturbed [5-7]. POAG is a neurodegenerative disease characterized by abnormal cupping of the optic nerve head and corresponding visual field defects that cannot be explained by other diseases, and with a normal open anterior chamber of the eye [8]. A major risk factor for POAG is an increased intraocular pressure (IOP) [9,10]. Proteomic analysis of POAG AH showed an increased level of total protein content and also the presence of larger and potentially harmful molecules compared to control AH [5-7]. This changed AH composition in POAG may ultimately affect the outflow facility of the TM via intrinsic molecular and structural changes, which will result in a decreased outflow and an increased IOP. The fourth aim of our study was to look for expression of POAG disease genes in the CBE, and we hypothesize about their potential role in the CBE in POAG when these genes are mutated.

The only proven therapy of POAG is lowering of the IOP. Drugs that lower the IOP target the CBE to decrease AH production or target the TM to increase AH outflow. With our newly formed hypothetical molecular model of AH production in the CBE (first aim of this study), we finally looked for potentially new targets to change the AH production with already existing drugs.

## Methods

### Ethics statement

This study was performed in agreement with the declaration of Helsinki on the use of human material for research. The human post-mortem donor eyes were obtained from the Corneabank, Beverwijk, the Netherlands. The Corneabank obtained permission (informed consent) from the donors for enucleation of the eyes and to use the eye for scientific purposes after removal of the cornea. All data were analyzed anonymously.

### Tissue sampling, RNA processing and microarray

We performed gene expression analysis on separately laser dissected NPE (n = 7) and PE (n = 7) cells (only pars plicata of the CBE) with microarray studies. For detailed description of donor eye selection, tissue sampling, RNA processing and microarray performance, we refer to our previously published paper [11]. In short, we selected seven healthy human donor eyes that were snap frozen. From 20 μm cryosections, the NPE and PE were separately cut out with laser dissection microscopy (PALM Carl Zeiss, Microlaser Technologies AG, Germany). Cresyl Violet staining was used to distinguish NPE (Merck, Frankfurt, Germany, art.5235). After selection of NPE and PE cells, RNA was isolated, amplified and labeled. Microarrays were performed against common reference sample (RPE/choroid RNA), in order to compare NPE and PE and normalize the data. The total gene expression datasets can be found in Gene Expression Omnibus database (GSE37957).

### Data analysis I: construction of sub-datasets

From the gene expression studies on the (N)PE, we ranked the genes by expression level and assigned percentile ranks (P) [12]. Next, we formed four sub-datasets: high expression (expression >90^th^ P), moderate expression (50-90^th^ P), low expression (10-50^th^ P) and very low expression (<10^th^ P). This means, for example, that a gene in the high expression sub-dataset has an expression intensity that falls into the highest 10% intensity values of the microarray. We also performed a statistical comparison between NPE and PE and stated gene expression statistically significant different between NPE and PE when p-value < 0.01 (correction for multiple testing; *Significant Different sub-dataset*). Finally, we determined the signature genes of NPE and PE, based on the selection criteria of fold-change >2.5 and p-value < 0.01 between NPE and PE.

From these different gene expression analyses, we studied the molecular background of AH production. In previous functional and physiological studies of *in vitro* and *in vivo* animal models of the CBE, ion channels that are involved in the AH production were widely studied. In a review of Civan and coworkers [2], these findings are put together in a model of AH production. We now looked for the gene expression level (high, moderate, low and very low) of the genes that code for these ion-channels. We also checked whether genes were a signature gene for one epithelial layer. We listed all the gene expression information of these ion-channel coding genes in Additional file 1: Table S1 and in Table 1 we presented genes with moderate or high expression level in at least one epithelial layer.

**Table 1:** Human CBE gene expression profiles for ion channels implicated in AH production

**Ion channel**	**Gene name**	**NPE**	**PE**	**SIG**
Water channel	AQP1^*^	H	H	
	*AQP2*	M	M	
	*AQP5*	L	M	
	AQP11^*^	M	M	
Na^+^/K^+^ ATPase	ATP1A1^*^	H	H	
	ATP1A2^*^	H	H	
	*ATP1A3*	M	M	
	*ATP1A4*	M	M	
	ATP1B1^*^	H	H	PE
	ATP1B2^*^	H	H	
	ATP1B3^*^	H	H	
Cl^-^ channel	*CLCN2*	L	M	
	*CLCN3*	M	M	
	*CLCN6*	M	M	
	*CLCN7*	M	M	
	CLIC1^*^	H	H	
	*CLIC2*	L	M	
	CLIC4^*^	H	H	
	CLIC6^*^	M	H	
Gap junction	GJA1^*^	H	H	
	GJA4^*^	M	M	PE
K^+^ channel	KCNA5^*^	L	M	
	*KCNAB1*	M	M	
	*KCNB1*	M	M	
	KCNB2^*^	L	M	PE
	KCND2^*^	L	M	PE
	KCND3^*^	M	M	
	*KCNE1*	M	M	
	*KCNE3*	H	M	
	*KCNE4*	L	M	
	*KCNG1*	M	M	
	*KCNJ12*	M	M	
	KCNJ13^*^	H	H	
	*KCNJ14*	M	M	
	*KCNJ2*	M	M	
	KCNJ8^*^	M	M	
	*KCNK1*	M	M	
	*KCNK13*	M	M	
	*KCNK15*	M	M	
	*KCNK3*	M	L	
	*KCNK4*	L	M	
	*KCNK7*	M	M	
	KCNQ1^*^	M	M	
	KCNS3^*^	M	M	
	*KCNV2*	M	M	
Na^+^ channel	*SCN2B*	M	M	
	*SCN3B*	M	M	
	SCN4B^*^	H	H	
Na^+^/2Cl^-^/K^+^ co-transport	*SLC12A2*	M	M	
Cl^-^/HCO3^-^ exchanger	SLC4A2^*^	L	M	
	*SLC4A3*	M	M	
Na^+^/HCO3^-^ symport	*SLC4A4*	M	M	
	*SLC4A7*	M	M	
Na^+^/H^+^ exchanger	*SLC9A5*	M	M	
	*SLC9A6*	M	L	
	*SLC9A9*	M	M	

Expression profiles of genes coding for ion channels implicated in aqueous humor (AH) production in healthy human non-pigmented (NPE) or pigmented epithelium (PE) of the ciliary body. The list of ion-channels involved in AH production is from Civan et al., 2004. We ranked the genes by expression level and assigned percentile ranks (P). Next, we formed four groups: high (H) expression (expression >90th P), moderate (M) expression (50-90th P), low (L) expression (10-50th P) and very low (VL) expression (<10th P). We also compared the NPE and PE expression data and indicated if genes were signature genes (SIG) of an epithelial cell layer (p-value < 0.01 and fold change >2.5). In this table, we only presented genes with moderate or high expression level in at least one epithelial layer. The total list of ion-channels and gene expression profiles is presented in Additional file 1: Table S1. Genes indicated with ^*^ are also present in *Transport sub-dataset* and indicated in Figure 2.

### Data analysis II: molecular machinery of AH production, CBE transport and (N)PE interaction

**Figure 1:**
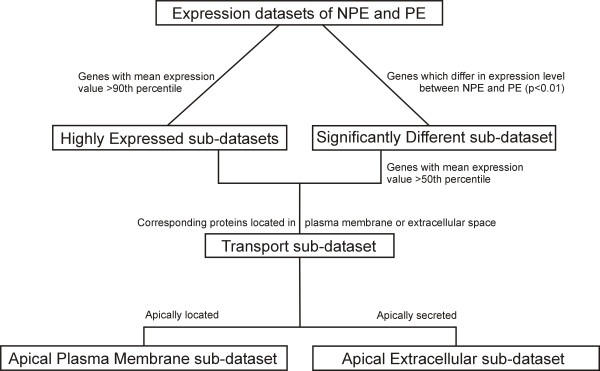
**Flow diagram.** Schematic overview of the subdivisions made in the gene expression data of the NPE and PE into different sub-datasets.

We further studied the molecular machinery of AH production, CBE transport and interaction of NPE and PE. We therefore created new sub-datasets. First, we combined the genes from the *Highly Expressed NPE and PE sub-datasets* (>90^th^ P) and the genes from the *Significantly Different sub-dataset* (with mean gene expression value higher than 50^th^ percentile), since we assumed that at least these genes are of biological importance for the (N)PE [12,13]. We took all Significantly Different expressed genes together, so without subdivision between NPE and PE, since it is difficult to attribute specific biological meaning in a set of up- or down regulated genes, without a base-line, to one of two specific (N)PE layers that are both healthy and from the same person (p-value<0.01 and fold-change >2.5). Still, we believe that these differences, without attributing them to a specific epithelial layer, are of biological meaning. We therefore choose to include the highly and significant different expressed genes together for future analysis. The cell specific genes were determined with much more strict criteria and stated as signature genes. The signature genes were than specifically attributed to PE or NPE. This approach was also previously used [11]. From this pool we selected those genes that coded, according to the Ingenuity knowledge database, for either plasma membrane or extracellular secreted proteins. Obviously, the entries from this selection are most likely to be involved in transmembrane transport mechanisms and interactions between two cell types compared with genes localized in the cytoplasm or nucleus. We named this new dataset, which contained highly expressed genes in the plasma membrane or extracellular space of the CBE, the *Transport sub-dataset* (flow diagram in Figure 1) (total *Transport sub-dataset* in Additional file 2: Table S2).

### Data analysis III: detailed analysis of the transport sub-dataset

We analyzed the Transport sub-dataset from five different points of view:

1) *In silico* hypothetical molecular model of the AH production: We translated the model of AH production from Civan and coworkers [2] into a hypothetical *in silico* molecular model of AH production based on our *Transport sub-dataset.* We specifically indicated the signature genes in the figure.

2) Transport mechanisms in the neuro-epithelia of the CBE: We studied all transporters and ion channels present in the *Transport sub-dataset* to infer possible specialized transport functions of the CBE. The signature genes of NPE or PE were specifically indicated. For conformation, we performed immunohistochemistry of two proteins of interest, caveolin-1 (CAV1) and clathrin heavy chain (CLTR). The methods of immunohistochemistry were described in detail elsewhere [11]. We used primary antibodies of Abcam (Cambridge, UK), for CAV1 a rabbit polyclonal (ab2910) and for CLTR also a rabbit polyclonal (ab21679).

3) Apical PE-NPE interaction and transport mechanisms: In order to study the possible molecular interaction PE and NPE, we selected from the *Transport sub-dataset* those genes which encoded proteins that are apically present in the plasma membrane or apically secreted into the extracellular space. We derived the protein localizations from the Ingenuity knowledge database and from Pubmed. We used the search term '*gene name* AND (apical OR basolateral OR subcellular location OR immuno location)’. We formed two new sub-datasets, named the *Apical Plasma Membrane sub-dataset* and *Apical Extracellular secreted sub-dataset* (flow-diagram in Figure 1). With these two newly formed sub-datasets, we analyzed functional interaction by exploring possible direct connections between the entries using Ingenuity’s tool 'Connect’, resulting in two functional molecular networks. The first one indicating the functional interaction between apical secreted molecules in the extracellular space between NPE and PE and the second one indicating the transport molecules in the apical plasma membrane that are connected to the apical secreted molecules from the former network. Entries connected to themselves have a circle on top; entries that did not have a connection with another gene were removed (detailed description of Ingenuity see http://www.ingenuity.com and [11]). Yellow symbols indicate PE signature genes.

4) CBE transport and possible implications for POAG: Based on the list of (candidate) POAG disease genes that we recently selected (reviewed by [14]), we looked in our *Transport sub-dataset* for presence of these (candidate) POAG disease genes.

5) Possible pharmacological targets for AH production altering drugs within the CBE. Within the *Transport sub-dataset* we looked for genes that code for targets of known AH production lowering drugs. We also looked in the hypothetical molecular model for AH production that we built (first aim) for drugs that target on proteins in this model. These drugs might be of interest for future POAG medication strategies to alter/decreased AH production.

## Results

### 1. *In silico* hypothetical molecular model of the AH production

**Figure 2:**
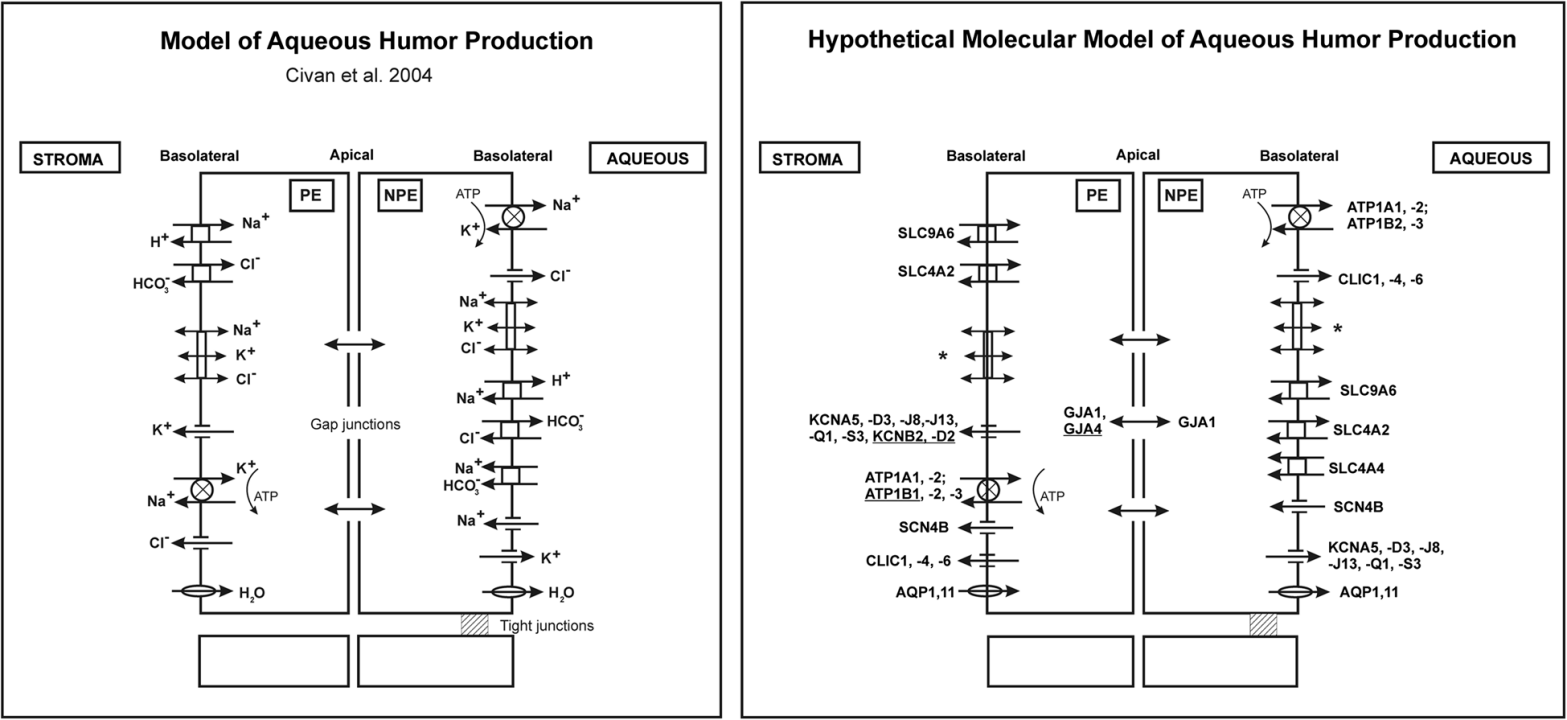
**Hypothetical molecular model of aqueous humor production.** On the left, there is the physiological model of aqueous humor (AH) production of Civan and coworkers [2]. This model includes the different ion-channels involved in the AH production. On the right, there is hypothetical *in silico* molecular model of AH production. This model is based on our gene expression data of the ciliary body epithelia (CBE) and our strict selection criteria of the 10% highest and significant different expressed genes of the non-pigmented (NPE) and pigmented epithelia (PE) (all together in the *Transport sub-dataset*; see Methods). For the Na^+^/K^+^/2Cl^-^ symporter (coded by *SLC12A1* and *SLC12A2*) we did not found a coding gene in the *Transport sub-dataset* (indicated with * in the model). Genes coding for ion-channels with moderate expression levels (for example *SLC12A2*) can be found in Table 1. The underlined genes (*ATP1B1, GJA4, KCNB2, KCND2*) are signature genes of the PE compared to the NPE (fold change >2.5 and p-value < 0.01; see Methods section).

Twenty four highly expressed genes in our *Transport sub-dataset* coded for ion-channels involved in AH production*.* Furthermore, 32 additional ion-channel coding genes were moderately expressed in the NPE and/or PE (Table 1). Of all these genes, four were signature genes of the PE compared the NPE, namely *ATP1B1, GJA4, KCNB2,* and *KCND2*. We translated the model of AH production of Civan and coworkers [2] into a hypothetical *molecular* model of AH production in which we included the genes of the *Transport sub-dataset* (Figure 2).

### 2. Transport mechanisms in the neuro-epithelia of the CBE

To determine which CBE highly expressed genes could ultimately be involved in the composition of the AH, we assessed the expression of all transporters and (cat-)ion channels and their specific substrate in the *Transport sub-dataset.* Total list is outlined in Table 2. Different groups of transporters can be distinguished.

**Table 2:** **All transporters in the *Transport sub-dataset *** **of the ciliary body epithelia**

**Symbol**	**ID**	**Symbol**	**ID**	**Symbol**	**ID**
A2M	NM_000014	GJA4^a^	NM_002060	SLC22A4	NM_003059
ABCA1^a^	NM_005502	GPIHBP1	NM_178172	SLC22A5	NM_003060
ABCA4	NM_000350	GPM6A	NM_201591	SLC23A2^a^	NM_203327
ABCA5	NM_018672	GRIN2C	NM_000835	SLC24A3	NM_020689
ABCA7	NM_019112	GRIN3A	NM_133445	SLC26A7^a^	NM_052832
ABCB1	NM_000927	HCN2	NM_001194	SLC27A1	NM_198580
ABCC1^a^	NM_019862	HEPH	NM_014799	SLC29A4	NM_001040661
ABCC5	NM_005688	IGFBP7	NM_001553	SLC2A1	NM_006516
ABCC9	NM_020297	ITGAV^a^	NM_002210	SLC2A4	NM_001042
ABCG2	NM_004827	KCNA5	NM_002234	SLC31A2	NM_001860
ANO2	NM_020373	KCNB2^a^	NM_004770	SLC35G1	NM_153226
ANO6	NM_001025356	KCND2^a^	NM_012281	SLC38A1	NM_030674
ANXA2	NM_001002857	KCND3	ENST00000369697	SLC38A2	NM_018976
ANXA4	NM_001153	KCNJ13	NM_002242	SLC38A9	NM_173514
ANXA5	NM_001154	KCNJ8	NM_004982	SLC39A14	NM_015359
ANXA7	NM_004034	KCNMB4	NM_014505	SLC39A8	NM_022154
APOE	NM_000041	KCNQ1	NM_000218	SLC3A2	NM_001012661
AQP1	NM_198098	KCNS3	NM_002252	SLC40A1^a^	NM_014585
AQP11	NM_173039	LDLR	NM_000527	SLC43A3	NM_199329
ATP13A1	NM_020410	MAL	NM_002371	SLC45A2	NM_016180
ATP13A3	ENST00000310773	NRXN3	NM_004796	SLC4A4	NM_003759
ATP1A1	NM_000701	ORAI1	NM_032790	SLC5A6	NM_021095
ATP1A2	NM_000702	PDPN	NM_006474	SLC6A13	BC020867
ATP1B1^a^	NM_001677	PKD2	NM_000297	SLC6A15^a^	NM_182767
ATP1B2	NM_001678	RAMP1^a^	NM_005855	SLC6A20	NM_020208
ATP1B3	NM_001679	RBP1	NM_002899	SLC7A5	NM_003486
ATP2B3	NM_021949	RBP4	NM_006744	SLC7A7	NM_003982
ATP2B4	NM_001001396	REEP5	NM_005669	SLC7A8	NM_182728
ATP9A	NM_006045	SCARB1	NM_005505	SLC9A3R1	NM_004252
BEST1	NM_004183	SCN4B	NM_174934	SLC9A6	NM_001042537
CACNA1C	NM_000719	SCN8A	AK091315	SORL1	NM_003105
CAV1	NM_001753	SERINC1	NM_020755	STXBP3	NM_007269
CDH23	NM_022124	SLC12A4	NM_005072	SV2B	NM_014848
CLIC4	NM_013943	SLC12A7	NM_006598	SYPL1	NM_182715
CLIC6	NM_053277	SLC13A3	NM_001011554	SYT13	NM_020826
CLNS1A	NM_001293	SLC13A4	NM_012450	TF	NM_001063
CLTA	NM_007096	SLC16A2	NM_006517	TM9SF2	NM_004800
CLTC	NM_004859	SLC16A3	NM_001042422	TNFAIP1	NM_021137
CNGB3	NM_019098	SLC16A6	NM_004694	TPCN1	AB032995
ECM1	NM_004425	SLC19A2	NM_006996	TRAK2	NM_015049
EXOC7	NM_001013839	SLC1A3	NM_004172	TRPM1	NM_002420
FOLR1	NM_016725	SLC1A4	NM_003038	TRPM3	NM_206948
FXYD6^a^	NM_022003	SLC1A7	NM_006671	TTR	NM_000371
GABRR1	NM_002042	SLC20A1	NM_005415	VAT1	NM_006373
GJA1	NM_000165	SLC20A2	NM_006749	VTI1B	NM_006370

Legend: *Transport sub-dataset* of the CBE contains all NPE and PE highly expressed and significantly different expressed genes, whose protein products are located in the plasma membrane or secreted in the extracellular space. Genes indicated with ^a^ are signature genes of the PE. We did not identify signature genes of the NPE that code for transporters. Abbreviations of gene names are according to standard abbreviations used in Genbank.

#### Vesicle mediated transporter gene expression in CBE: caveolin and clathrin

We identified many different entries in our *Transport sub-dataset* involved in endo- and exocytosis as well as vesicle mediated transport (*ANXA2, -4, -5, -7, CAV1, CLTA, CLTC, EXOC7, SORL1, STXBP3, SV2B, SYPL1, SYT13, VAT1, and VTI1B*).

**Figure 3:**
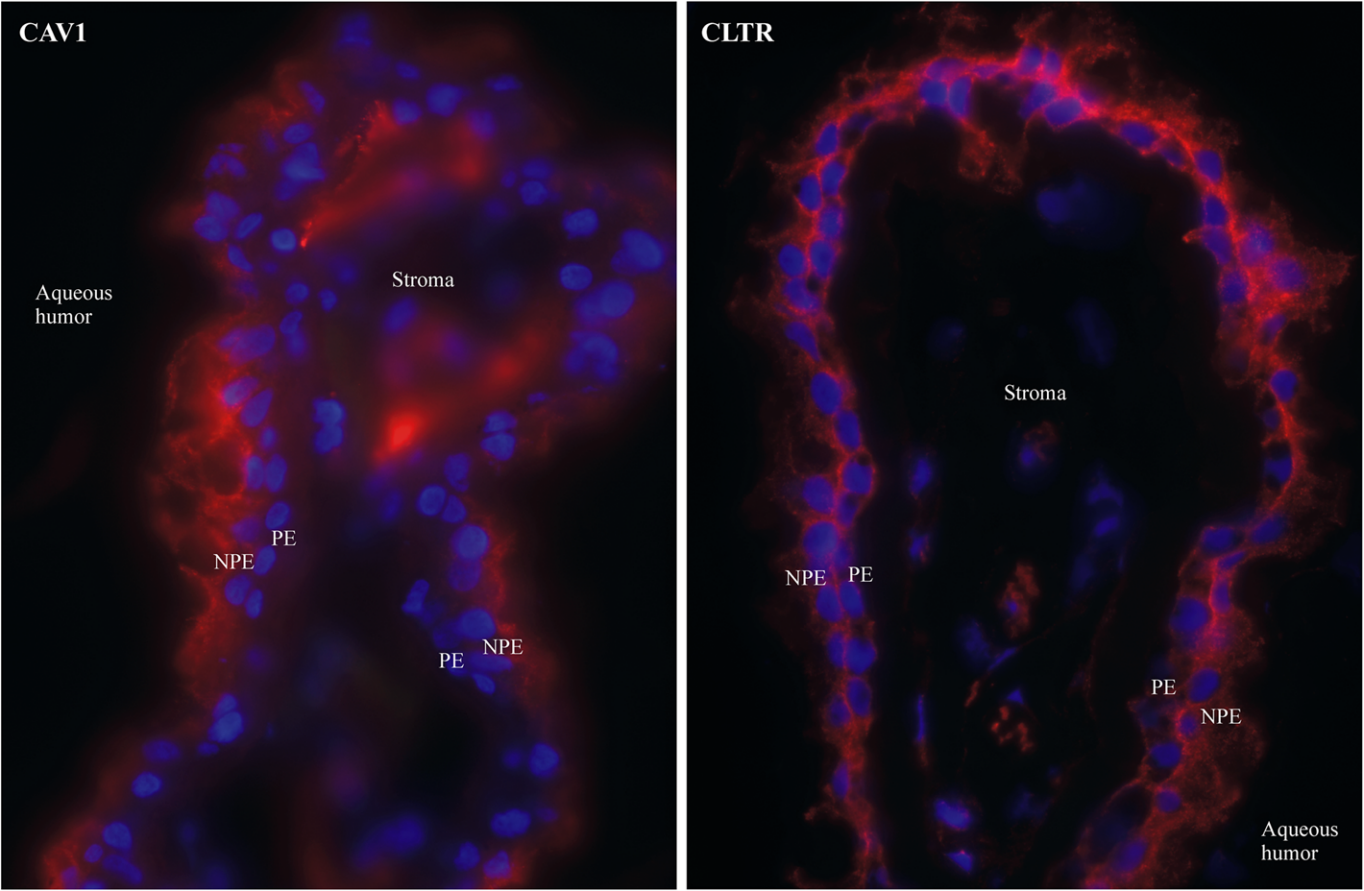
**Immunohistochemistry of caveolin 1 and clathrin in the ciliary body.** Immunofluorescence staining for caveolin 1 (CAV1) and clathrin (CLTR) in human ciliary body sections. Our gene expression data show that *CAV1* is significantly different expressed between NPE and PE (p-value < 0.01), with higher expression in NPE and all CLTR coding genes (*CLTA, CLTB* and *CLTC*) are highly (>90^th^ P) expressed in both NPE and PE. In this immunofluorescence picture, CAV1 is clearly present in the non-pigmented epithelium (NPE) and possibly also in the pigmented epithelium (PE). We found CLTR in both NPE and PE, especially in the apical plasma membrane. Both CAV1 and CLTR were found in the vascular endothelium of the stroma. Negative controls were for both proteins negative (not shown). Legend: blue = dapi = cell nucleus; red = cy3 = protein of interest.

Using immunohistochemistry, we confirmed the presence of caveolin-1 (CAV1) and clathrin heavy chain proteins (CLTR) in the CBE (Figure 3). More specifically, we found strong staining of CAV1 in the NPE and possible light staining in the PE. Furthermore, we observed strong staining of CLTR in both the NPE and PE of the CBE, especially in the apical plasma membrane.

#### Specific transporter activity in CBE: hormones, vitamins, metabolism, metal ions

We found a number of genes coding for neuro-endocrine transporters, like glutamate transporters (*SLC1A3, SLC1A4, SLC1A7*), glutamate-gated ion channels (*GRIN2C, GRIN3A*), GABA transporter (*SLC6A13*) and transporters of thyroid hormone (*SLC16A2, TTR*), thiamine (*SLC19A2*), vitamin C (*SLC23A2;* signature gene of PE), lipids (*SLC27A1, APOE, LDLR, SORL1, and SCARB1*) and glucose (*SLC2A1, SLC2A4*). We also identified genes coding for transporters of zinc (*SLC39A14, SLC39A8*), copper (*SLC31A2*) and iron (*SLC40A1* (signature gene of PE)*, TF, HEPH*) in the *Transport sub-dataset.*

#### Transporter activity in CBE: organic anions and ions (other than in the hypothetical molecular model of AH production)

We identified high expression of several genes coding for ABC transporters (*ABCA1, ABCA4, ABCA5, ABCA7, ABCB1, ABCC1, ABCC5, ABCC9* and *ABCG2*), responsible for organic anion transport. *ABCA1* and *ABCC1* are signature genes of the PE when compared to the NPE. Moreover, we found genes coding for phosphate transporters (*SLC20A1, SLC20A2*), carnitine transporters (*SLC22A4, SLC22A5*), and amino acid transporters (*SLC38A1, SLC6A15* (signature gene of PE)*, SCL7A5, SLC7A7, SLC7A8*). Next, we found genes coding for other ATP-transporters than that were already attributed for the AH production, namely *ATP2B3, ATP2B4, ATP13A1, -3,* and *ATP9A*, and genes coding for K^+^/Cl^-^ transporters (*SLC12A4, SLC12A7*) and the Na^+^/K^+^/Ca^2+^ exchanger (*SLC26A7,* signature gene of PE).

### 3. Apical PE-NPE interaction and transport mechanisms

#### Molecular interactions in the extracellular space between the (N)PE apical sides

**Figure 4:**
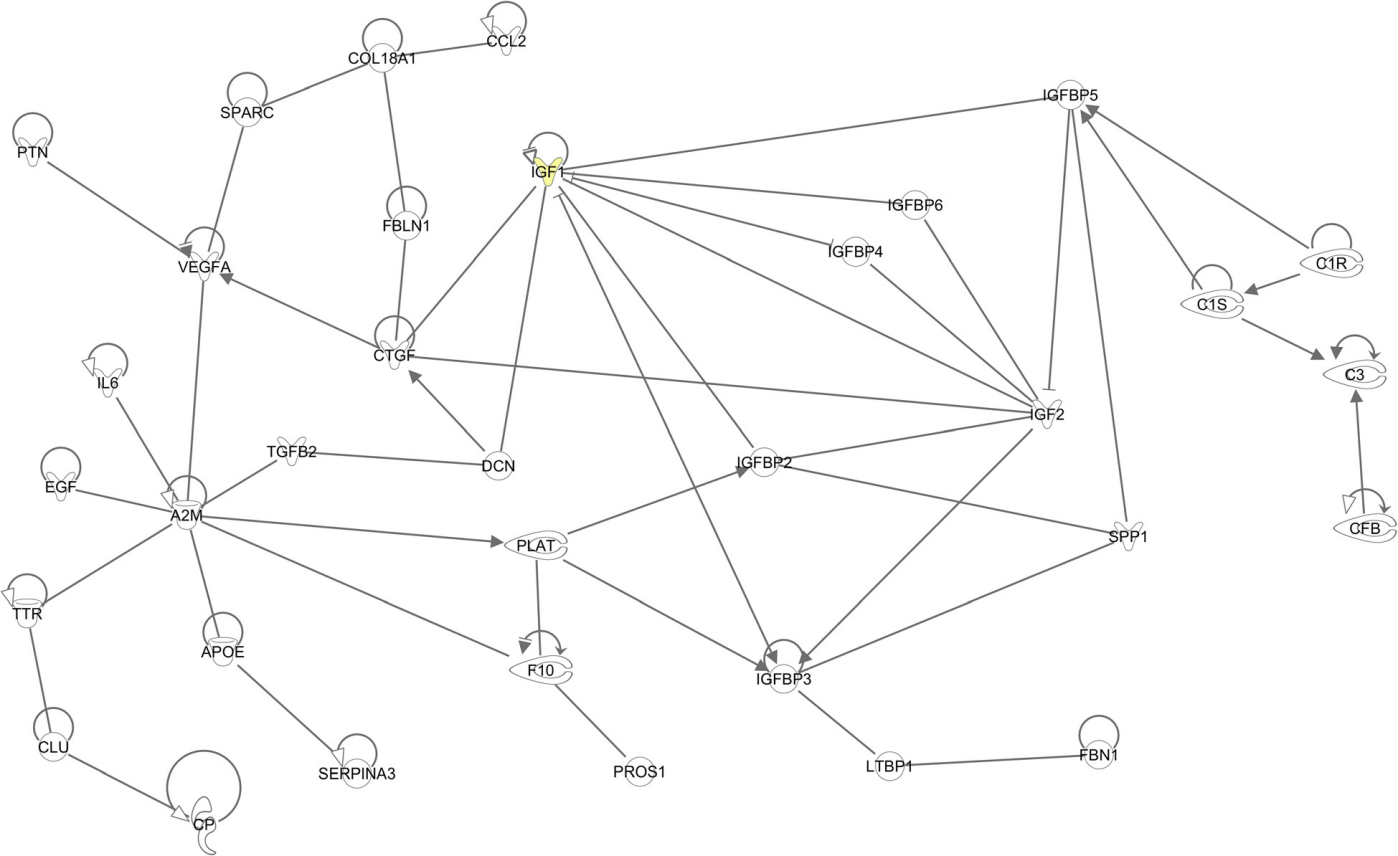
**Molecular interactions in the apical extracellular space of the pigmented (PE) and non-pigmented epithelium (NPE).** Molecular network build in Ingenuity (http://www.Ingenuity.com), representing entries that are highly expressed and secreted in the apical extracellular space between NPE and PE. The top functions assigned to this network by Ingenuity were “Neurological disease, psychological disorders and cellular movement”. This network is contains many entries involved in matrix assembly and cytoskeleton organization (*COL18A1, CTGF, DCN, FBN1, FBLN1, LTBP1, SPARC,* and *SPP1*) and molecules involved in growth and proliferation (*CTGF, EGF, IGF1, IGF2, IGFBP2-6, LTBP1, PTN, TGFB2* and *VEGFA*). *IGF1* is a signature gene of the PE (yellow symbol). Together, these molecules are likely to be involved in the local turnover of the ECM between the PE-NPE layers of the CBE. We also identified several entries within the PE-NPE apical extracellular space of the CBE that are involved in amyloid-beta metabolism and plaque formation (*A2M, APOE, CLU, SERPINA3,* and *TTR*). Legend of different lines: Circles on top indicate direct relationships of entries with itself*.* Solid lines indicate that binding of the two proteins occur, arrows indicate that the first protein interfere with the expression or activity of the second protein.

We explored the potential functional relationships between the predicted secreted entries of the *Apical Extracellular Secreted sub-dataset* (Figure 4). This network represents potential molecular interactions taking place in the extracellular space between the NPE and PE. The top functions assigned to this network by Ingenuity were “Neurological disease, psychological disorders and cellular movement”. The network contained 34 genes of a total of 67 (51%) secreted genes in the (N)PE apical extracellular space. Interestingly, this network contained many entries involved in matrix assembly and cytoskeleton organization (*COL18A1, CTGF, DCN, FBN1, FBLN1, LTBP1, SPARC,* and *SPP1*). Indeed, these proteins may partly fill the extracellular space between the apical membranes of the NPE and PE, as was previously shown for FBN1 by Gabriel and coworkers [15]. We also found genes coding for components of the classical complement cascade (*C1R, C1S, C3,* and *CFB*), the coagulation pathway (*F10, PLAT* and *PROS1*) and immune and/or inflammatory responses (*CCL2* and *IL6*) and we identified many genes coding for a range of growth factors (*CTGF, EGF, IGF1, IGF2, IGFBP2-6, LTBP1, PTN, TGFB2* and *VEGFA*). Finally, we identified several entries apparently involved in amyloid beta metabolism and plaque formation (*A2M, APOE, CLU, SERPINA3,* and *TTR*).

#### Molecular interactions in apical transport of the CB

**Figure 5:**
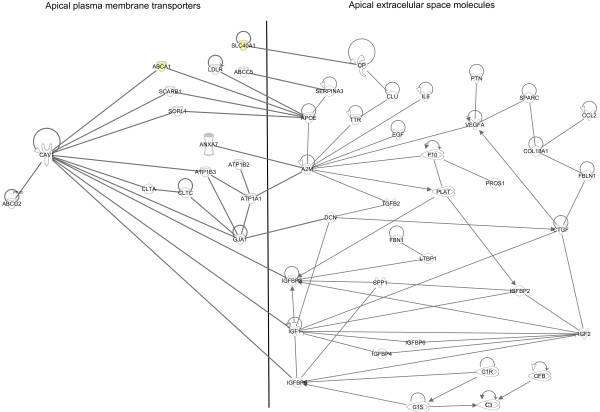
**Molecular interactions in apical transport of the ciliary body epithelia.** Molecular network build in Ingenuity (http://www.Ingenuity.com), representing possible transporters in the apical plasma membranes of the pigmented and non-pigmented epithelia that transport the apical extracellular excreted entries outlined in Figure 4. The top functions assigned by Ingenuity to this network were “Neurological disease, psychological disorders and cell-to-cell signaling and interaction”. The major transport mechanisms to which these genes were connected were vesicle-mediated transporters (*ANXA7, CAV1, CLTA, CLTC,* and *SORL1)* and lipid (*ABCA1, LDLR* and *SCARB1)* and xenobiotic (*ABCC5* and *ABCG2*) transporters. Two PE signature genes, *ABCA1* and *SLC40A1* (yellow symbols) are involved in this network. Legends of different lines: see Figure 4.

We were interested in the transport mechanisms of the entries in the apical extracellular space of the NPE and PE. Therefore, we explored *in silico* potential functional relationships between the genes coding for transporters in the *Apical Plasma Membrane sub-dataset* and the network described above. The resulting network is displayed in Figure 5. The top functions assigned by Ingenuity to this network were “Neurological disease, psychological disorders and cell-to-cell signaling and interaction”. This network showed direct interactions of 15 transporter genes (from a total of 50 genes; 30%) in the apical plasma membrane with the apical extracellular secreted molecules. Clearly, several entries involved in vesicle-mediated transport were connected to the extracellular secreted entries, namely *ANXA7, CAV1, CLTA, CLTC,* and *SORL1.* Furthermore, we identified several genes in the apical plasma membrane that codes for proteins involved in lipid transport (*ABCA1* (signature gene of PE)*, LDLR* and *SCARB1*), iron transport (*SLC40A1*; signature gene of PE) and xenobiotic/drug transport (*ABCC5* and *ABCG2*) that are connected to molecules in the apical extracellular space of the CBE.

### 4. CBE transport and possible implications for POAG

In our *Transport sub-dataset* we found seven genes that were previously associated with POAG, namely *APOE, CAV1, COL8A2, EDNRA, FBN1, RFTN1* and *TLR4*. None of these genes were signature genes of the NPE or PE.

### 5. Possible pharmacological targets for AH production altering drugs within the CBE

The three classes of drugs that can lower AH production are adrenergic agonists, carbonic anhydrase (CA) inhibitors and beta blockers. Within our *Transport sub-dataset* we found high expression of *CA4* and *CA14* in the plasma membrane of the CBE.

We also looked closer to the ion-channels in the hypothetical model of AH production (Figure 2) for potentially new drugs that might target these channels. For the Na+/K + ATPase (coded by *ATP1A1* and *ATP1A2)* there are several inhibitors, namely digoxin, omeprazole, ethacrynic acid and perphenazine. Two sodium channels coded by *SCN4B* and *SCN8A* genes are inhibited by riluzole, a drug used in ALS. Finally, the potassium channels coded by *KCNA5* and *KCNQ1* can be blocked by quinidine, amiodarone, tedisamil and flecainide (antianginal and antiarrhythmic drugs) or opened by nicorandil (frequently used against hypertension).

## Discussion

In order to shed light on the molecular mechanisms in the CBE related to AH production and composition, we analyzed our expression data of the NPE and PE from five different angles. First, we studied the expression profiles of the genes coding for all ion-channels involved in AH production and we built *in silico* a hypothetical molecular model for AH production. Next, we assessed putative transport mechanisms in the NPE and PE, including vesicle mediated and specialized transport. Third, we analyzed, *in silico,* several apical molecular networks present between the NPE and PE, amongst others with respect to transport and extracellular matrix molecules. Fourth, we identified a number of clues which suggest that the CBE plays an important (transport) role in POAG. And finally, we looked for existing and possible new pharmacological targets in the CBE which could potentially modify the AH production.

Our microarray approach and corresponding functional annotations have several limitations, which are extensively discussed elsewhere [12,13]. The gene expression datasets used in the current study were first roughly analyzed in a previous paper, which describes the general properties of the CBE [11]. The value of the current study is that we now analyzed these datasets in detail, primarily focused on the molecular machinery underlying transport mechanisms. Fortunately, the physiology of the transport mechanisms in the CBE has previously already been studied extensively in *in vitro* and *in vivo* models, and was leading for our molecular analysis and interpretation.

Obviously, the amount of (combination of old and) new data presented in this manuscript prohibits verification *in vitro* and *in vivo* models in one single study, let alone in this manuscript.

Finally, it must be taken into account that we used very strict selection criteria for our *Transport sub-dataset.* Therefore, the genes that we selected for further future analysis in this manuscript may not cover *all* genes that are involved in the transport and AH production functions of the CBE. It is not unlikely that there is functional redundancy, and that several similar genes may code for more or less effective additional transport proteins, or that more – yet to be identified – proteins may be involved in AH production. On the other hand, because of our strict selection criteria, the genes that *are* present in this model, are likely to be truly involved in the molecular machinery of AH production and CBE transport mechanisms.

### 1. *In silico* hypothetical molecular model of the AH production

We build a hypothetical molecular model of AH production based on the gene expression data of (the ion-channels in) the CBE (Figure 2). We selected genes under strict selection criteria in the *Transport sub-dataset*.

Due to these strict selection criteria, a number of additional genes or proteins, previously implicated in AH production, did not pop up in our present hypothetical molecular model (Figure 2). For example, for the Na^+^/K^+^/2Cl^-^ symporter (coded by *SLC12A1* or *SLC12A2*) we did not found a coding gene in our *Transport sub-dataset*, but these genes were moderately (*SLC12A2)* and lowly (*SLC12A1)* expressed in the (N)PE (Table 1).

### 2. Transport mechanisms in the neuro-epithelia of the CBE

The CBE expresses many genes coding for ion-channels and transporters at high levels. Due to their known substrate specificity of their corresponding proteins, we can also predict the biomolecules transported by these CBE entries. Consequently, our inventory, in combination with data from the literature, showed that the CBE most likely transports a wide variety of molecules, including cations, organic cations and anions, amino acids, glutamate, GABA, iron, copper, zinc, thyroid hormone, vitamin A, B and C, glucose, and fatty acids. Indeed, the majority of these ions and biomolecules were also found in previous proteomic studies of the AH, which suggests that the CBE is an important ocular entry point of these molecules [16-20].

Interestingly, we also observed high expression of many entries involved in vesicle mediated trans- or endocytosis. For example, we found high expression of *CAV1* and *CLTC* in the CBE and we confirmed their presence by immunohistochemistry (Figure 3). Caveolin and clathrin are involved in vesicle mediated endo-, exo- and transcytosis of large molecules through the cell [21-25]. Caveolin-mediated vesicles transport for example iron, transferrin, insulin, lipids, albumin, chemokines and pathogens [21]. Clathrin-mediated vesicles can transport nutrients, viruses, toxins, plasma membrane proteins and signaling receptors, including ß2-adrenergic receptors, CD4, insulin receptors, T-cell and B-cell receptors [24,25]. While caveolin transport is more or less a-specific, clathrin-mediated vesicle transport is only possible with adaptor proteins (receptors). We also found expression of several genes coding for other proteins involved in less specific vesicle transport, like *ANXA2, EXOC7, SORL1, STXBP3, SYT13, VAT1,* and *VTI1B*

In summary, our data, in combination with the literature, suggest that both general and specific vesicle mediated transport play a role in the transport of a range of biomolecules over the CBE neuroepithelia. Indeed, this was already suggested by a histochemical electron microscope study [26]. We hypothesize that these transport mechanisms may (also) be involved in AH composition, in particular with regard to the presence of large molecules.

### 3. Apical PE-NPE interaction and transport mechanisms

*In silico*, we assigned several interacting proteins to the extracellular space between the PE and NPE, where transported molecules from the blood to the AH are likely to pass. We found several proteins involved matrix assembly and cytoskeleton organization, a network of protease (inhibitors) and a group of proteins involved in cell growth and proliferation pathways. These molecules are likely to be involved in the local turnover of the ECM between the PE-NPE layers of the CBE. We also identified several entries within the PE-NPE apical extracellular space of the CB that are involved in amyloid-beta metabolism and plaque formation. The major transport mechanisms to which these entries were connected were vesicle-mediated transporters and lipid and xenobiotic transporters. The molecules within the PE-NPE extracellular space and their transporters may influence intrinsic filtration or secretion capabilities of the CBE. If disrupted by a genetic mutation, or altered by age-related oxidative stress, these molecules change and may affect the AH secretion and composition.

### 4. CBE transport and possible implications for POAG

We identified seven POAG candidate disease genes in our *Transport sub-dataset*. These genes were *APOE, CAV1, COL8A2, EDNRA, FBN1, RFTN1* and *TLR4.* We hypothesized that mutations in these genes might modify the AH production and composition via the CBE. Although the production rate of AH is hardly changed in POAG [27], the composition of AH does, with overall an increase of (large and harmful) proteins in POAG AH compared to controls [5-7]. The POAG disease genes that we found in our *Transport sub-dataset* code for structural components (FBN1, COL8A2), transporters (CAV1, APOE, RFTN1) and signaling molecules (CAV1, EDNRA), all potentially involved in AH production and composition. Further research is warranted to prove or reject their involvement in disturbed AH dynamics of the CBE during POAG. It would be of interest to study mouse models with mutations in these genes, measure IOP, optic nerve head characteristics and AH composition. It would also be of interest to study the effect of IOP-lowering drugs in animals with mutations in these target genes in comparison with controls.

### 5. Possible pharmacological targets for AH production altering drugs within the CBE

Last but not least, we annotated our molecular model (Figure 2, discussed above) with possible new therapeutic targets that might alter AH production. First, we found several drugs that block Na+/K + ATPases: digoxin, ethacrynic acid, omeprazol, and perphenazine. Digoxin has antiarrhythmic effects via myocytes and some older literature sources describe the lowering effect of digoxin on the IOP in human [28-30] and cats [31], probably due to blocking of Na^+^/K^+^-ATPase in the CBE. Ethacrynic is a diuretic drug and multiple animal studies already showed the IOP lowering effect of intracameral or topical administered ethacrynic acid in the eye, probably through increased AH outflow, but possibly also by decreased AH production [32-39]. The major disadvantage of athacrynic acid is the risk for (severe) corneal edema. Omeprazole is a proton pump inhibitor that inhibits gastric acid secretion in the stomach. In the literature, we found one clinical study in which treatment with omeprazole indeed lowered the IOP [40]. The drug perphenazine is an antagonist of dopamine receptors and has antiemetic and antipsychotic effects. We found no studies of perhanzine in relation to IOP altering effects.

Secondly, we found one drug that block sodium channels, namely riluzole. This drug is used in ALS. In the literature, we couldn’t find studies describing an effect on IOP with riluzole use.

Finally, we found several drugs that influence the activity of potassium channels. Quinidine, amiodarone, tedisamil and flecainide block potassium channels, while nicorandil opens them. Only for the latter drug, nicorandil, two studies exist on the IOP effects. Chiang and coworkers [41] found increased IOPs after nicorandil use in rabbits, whereas Chowdhury and coworkers [42] found an IOP lowering effect of nicorandil in rats.

In conclusion, we found possible new therapeutic targets for AH production modification in the CBE. These targets are of interest, since IOP lowering is currently the only proven therapy for POAG and current medical treatment modalities often fail to reduce the IOP sufficiently. More research into these new targets might result in new treatment options for POAG, which may make surgery, with its side effects and risks, less often needed. Extensive studies in animal models are warranted to study potential beneficial effects of these drugs on the IOP and their potential harmful side effects.

## Conclusions

The transport mechanisms in the CBE are numerous, ranging from extensively studied specific ion channels and pumps that form the basis of AH production, to more generalized transport functions like vesicle mediated transport. The CBE transports (neuro-) endocrine and metabolic particles and is involved in specific apical transport of ECM proteins and growth factors. Taken together, these transport mechanisms are important in determining the AH production rate and composition. In POAG, the composition of the AH is changed and several POAG disease gene are highly expressed, and their corresponding proteins localized in the CBE plasma membrane or extracellular space. We propose that mutations in these POAG genes cause disturbances in the AH composition by the CBE, resulting in a changed AH content and disturbed outflow facility by the TM. Finally, we found possible new therapeutic targets for AH production modification in the CBE.

## Abbreviations

AH: Aqueous humor; CBE: Ciliary body epithelia; IOP: Intraocular pressure; NPE: Non-pigmented epithelium; PE: Pigmented epithelium; POAG: Primary open angle glaucoma; TM: Trabecular meshwork.

## Competing interests

The authors declare that they have no competing interests

## Authors' contributions

SFJ carried out the laboratory work, performed the bioinformatic analysis and drafted the manuscript. TGG participated in the design of the study and the manuscript. PJS provided bioinformatics software and background knowledge. NMJ participated in the design of the study and the manuscript. AAB performed bioinformatic analysis and drafted the manuscript. All authors read and approved the final manuscript.

## Supplementary Material

Additional file 1: Table S1 Total human CBE gene expression profile for AH production. Click here for file

Additional file 2: Table S2 Total Transport sub-dataset. Click here for file
